# The Primary Care and Environmental Health e-Learning Course to Integrate Environmental Health in General Practice: Before-and-After Feasibility Study

**DOI:** 10.2196/56130

**Published:** 2024-05-09

**Authors:** Jean-Baptiste Tostain, Marina Mathieu, Agnès Oude Engberink, Bernard Clary, Michel Amouyal, Béatrice Lognos, Pascal Demoly, Isabella Annesi-Maesano, Grégory Ninot, Nicolas Molinari, Arnaud Richard, Maha Badreddine, Claire Duflos, Francois Carbonnel

**Affiliations:** 1 Desbrest Institute of Epidemiology and Public Health University of Montpellier National Institute for Health and Medical Research Montpellier France; 2 Departement of General Practice University of Montpellier Montpellier France; 3 Clinical Research and Epidemiology Unit Centre Hospitalier Universitaire de Montpellier Montpellier France; 4 Maison de Santé Pluriprofessionnelle Universitaire Avicennne Cabestany France; 5 Service de Pneumologie, Allergologie et Oncologie Thoracique Centre Hospitalier Universitaire de Montpellier Montpellier France; 6 University of Toulon Toulon France; 7 Department of Pedagogical Engineering and Audiovisual Production Faculty of Medicine University of Montpellier Montpellier France

**Keywords:** environmental health, medical education, One Health, environment, environmental, eLearning, e-learning, remote, learning, online learning, primary care, satisfaction, awareness, behavioral, behavior change, questionnaire, survey, course, educational, teaching, GP, general practice, general practitioner

## Abstract

**Background:**

Environmental and behavioral factors are responsible for 12.6 million deaths annually and contribute to 25% of deaths and chronic diseases worldwide. Through the One Health initiative, the World Health Organization and other international health organizations plan to improve these indicators to create healthier environments by 2030. To meet this challenge, training primary care professionals should be the priority of national policies. General practitioners (GPs) are ready to become involved but need in-depth training to gain and apply environmental health (EH) knowledge to their practice. In response, we designed the Primary Care Environment and Health (PCEH) online course in partnership with the Occitanie Regional Health Agency in France. This course was used to train GP residents from the Montpelier-Nimes Faculty of Medicine in EH knowledge. The course was organized in 2 successive parts: (1) an asynchronous e-learning modular course focusing on EH knowledge and tools and (2) 1 day of face-to-face sessions.

**Objective:**

This study assessed the impact of the e-learning component of the PCEH course on participants’ satisfaction, knowledge, and behavior changes toward EH.

**Methods:**

This was a pilot before-and-after study. Four modules were available in the 6-hour e-learning course: introduction to EH, population-based approach (mapping tools and resources), clinical cases, and communication tools. From August to September 2021, we recruited first-year GP residents from the University of Montpellier (N=130). Participants’ satisfaction, knowledge improvements for 19 EH risks, procedure to report EH risks to health authorities online, and behavior change (to consider the possible effects of the environment on their own and their patients’ health) were assessed using self-reported questionnaires on a Likert scale (1-5). Paired Student *t* tests and the McNemar *χ^2^* test were used to compare quantitative and qualitative variables, respectively, before and after the course.

**Results:**

A total of 74 GP residents completed the e-learning and answered the pre- and posttest questionnaires. The mean satisfaction score was 4.0 (SD 0.9) out of 5. Knowledge scores of EH risks increased significantly after the e-learning course, with a mean difference of 30% (*P*<.001) for all items. Behavioral scores improved significantly by 18% for the participant’s health and by 26% for patients’ health (*P*<.001). These improvements did not vary significantly according to participant characteristics (eg, sex, children, place of work).

**Conclusions:**

The e-learning course improved knowledge and behavior related to EH. Further studies are needed to assess the impact of the PCEH course on clinical practice and potential benefits for patients. This course was designed to serve as a knowledge base that could be reused each year with a view toward sustainability. This course will integrate new modules and will be adapted to the evolution of EH status indicators and target population needs.

## Introduction

The World Health Organization (WHO) defines environmental health (EH) as “those aspects of human health, including quality of life, that are determined by the physical, chemical, biological, social, psychosocial and aesthetic factors in our environment” [1]. According to the WHO, environmental exposures are responsible for 12.6 million deaths each year worldwide and contribute to cancer, cardiovascular, respiratory, neurological, and infectious diseases [2]. Environmental and behavioral factors are estimated to be the cause of approximately one-quarter of deaths and chronic diseases (ie, 20% of cancers, 30% of cardiovascular diseases, and 35% of respiratory diseases). In Europe, over 1.4 million premature deaths are caused by polluted environments [2]. The Food and Agriculture Organization of the United Nations, United Nations Environment Programme, WHO, and World Organisation for Animal Health have set a goal of creating healthier environments by 2030 in working together through the One Health initiative [3]. The French High Council for Public Health reiterated that achieving this goal was a political, financial, and scientific priority. However, inequalities in health are widening in France, mainly because of underresourced and poorly organized prevention and primary care systems [4].

Involving primary care in EH is an important support in the training of health care professionals. This training is a priority issue and has been reiterated as such in the fourth French national EH plan [5]. General practitioners (GPs) are clearly ready to become involved in this initiative; 93% of GPs believe that they should play an important role in informing their patients about EH risks [6], whereas only 55% feel they can respond to this topic, and 75% of them have not been trained on the topic, an observation shared by other disciplines [6-9]. The French national agency for continuing professional development has set understanding EH issues as one of its priorities for 2023-2025, which represented less than 1% of its approved courses in 2022 [10]. Training GPs while they are still at university could be a worthwhile investment and help to overcome these difficulties, as 84% of French health students express a desire to be trained in this area [11]. In the United Kingdom, the integration of an EH course into medical school curricula has improved the ability of GPs to discuss EH with their patients [12]. The COVID-19 pandemic has encouraged the development of online (e-learning) courses [13]. An e-learning course in EH aimed at pediatric health professionals has already shown an average 30% increase in general knowledge, with a greater increase in subjects whose level of knowledge was initially low [14]. GPs appreciate this format for their continuing professional training, especially when combined with face-to-face teaching [15,16].

The Primary Care Environment and Health (PCEH; *SPES* in French for *Soins Primaires Environnement et Santé*) course was designed to train GP residents from the Montpelier-Nimes Faculty of Medicine in EH and, for this first year, teachers from the faculty’s Department of General Practice. The PCEH course was organized in 2 successive parts: (1) an asynchronous e-learning modular course focusing on EH knowledge and tools, and (2) 1 day of face-to-face sessions with lectures by EH experts in the morning and problem-solving workshops in the afternoon to support a thesis in EH. The e-learning was designed to serve as a knowledge base that could be reused each year, with a view to making the PCEH course sustainable.

The aim of this pilot study was to assess the impact of the EH e-learning on satisfaction, knowledge, and behavior of first-year GP residents.

## Methods

### Study Design

This was an interventional study based on a before-and-after, open, non-randomized, monocentric design. The study is reported per the guidelines for nonrandomized pilot and feasibility studies [17] and in line with the CONSORT (Consolidated Standards of Reporting Trials) 2010 checklist for reporting a pilot or feasibility trial (items pertinent to randomization were considered to be not applicable) [18].

To structure our inquiry, we relied on the Kirkpatrick model [19], which is a commonly used framework for evaluating learning. This model frames training on four levels: (1) *reaction*, (2) *learning*, (3) *behavior*, and (4) *results*. *Reaction* is the degree to which participants find the training favorable, engaging, and relevant to their jobs; *learning* is the degree to which participants acquire the intended knowledge, skills, attitude, confidence, and commitment based on their participation in the training; *behavior* is the degree to which participants apply what they learned during training when they are back on the job; and *results* is the degree to which targeted outcomes occur as a result of the training and the support and accountability package. A more recent version of this model added that the behavior level refers to “required drivers” or factors that increase the likelihood that people will retain and apply what they have learned in a given setting [20].

### Participants

The study population included first-year GP residents at the Montpellier-Nimes Faculty of Medicine. An email was sent to the potential participants, inviting them to identify themselves on Moodle, the learning management system of the University of Montpellier, to access the online training course. Only participants who completed the pre- and posttest questionnaires were included in the final analysis.

### e-Learning Intervention Content

The general objective of the e-learning course was to enable GPs to integrate EH risks for a given region into their practice. The course consisted of 4 modules, which are described in Table 1.

**Table 1:** Content of the e-learning Primary Care Environment and Health (PCEH) course.

Modules and learning objectives			Supports
**1. Introduction to EH^a^: Raising awareness of the impact of the environment on health**			
	Define EH and the exposome	Commented slideshow	
	Define an EH risk	8 videos (outdoor air, indoor environment, water, noise, UV, classified facilities and emitters, endocrine disrupters, vector-borne pathologies)	
	Describe current regulations on environmental risks	Interactive texts: modular courses with interactive sorting, labeled graphics, process, timeline, accordion blocks, and responsive quiz to enhance student feedback	
	Define a territorial EH diagnosis	Text document, illustrated with figures	
	Describe the fourth national EH plan	Commented slide show and, when relevant, examples of potential medical thesis topics	
**2. Population-based approach: Identifying the environmental risks of a population**			
	Differentiate population-based approach, individual approach, and situational analysis	Interactive texts	
	Identify an EH risk in an area using mapping tools (GEODES, Atlasanté, and SIRSE)	Interactive texts and videos	
	List the EH resources available to GPs^b^	Interactive texts	
	List the tools (institutional and noninstitutional) for assessing and analyzing the various risks associated with an area and a population (Occitanie territorial EH diagnosis)	Interactive texts and commented slideshows	
	Organize monitoring methods for the practice’s patients (Recosanté, ATMO, Pollen.fr, DGS-urgent)	Interactive texts	
	Describe how to report an EH risk event to health authorities	Interactive texts	
3. Integrating EH into my practice for my patients: Identify the environmental risks to which an individual is exposed in a clinical case, use tools to assess the various risks associated with an area and a population in an educational situation, include the patient’s opinion in defining an appropriate response to their EH risk			Three clinical cases based on 5-6 multiple-choice questions (exposure to lead, atmospheric pollution, and UV radiation) with immediate debriefing of the answers to help participants realize how far they had progressed and the practical usefulness of the tools proposed
**4. Communication: Promoting an EH intervention designed as part of PCEH training**			
	List the internal, institutional, and noninstitutional means of communication for promoting an EH intervention	Interactive texts	
	Analyze EH communication using the example of the Occitanie website of the Agence Régionale de Santé [21].	Video	

^a^EH: environmental health.

^b^GP: general practitioner.

Each module contained a hypertext link to the Rise360 software, which was interfaced with Moodle to generate interactive online courses. This platform can be used to display alternating slideshows, videos, audio, and interactive text in a fluid manner, which facilitates acquisition, creates an environment that involves the participants, and enhances attention span throughout the course [22]. The course lasted between 4 and 6 hours and served as a prerequisite for the compulsory face-to-face training course on September 9, 2021, which validated their participation for the GP curriculum.

### Data Collection

The assessment was carried out directly on Moodle using Microsoft Forms.

The pre- and posttest questionnaires contained 34 questions (see Multimedia Appendix 1). These questionnaires were created by the author team given the lack of an existing validated questionnaire for this population in this study context. The posttest questionnaire was available if all 3 first learning modules were completed, as the fourth module (Communication) had been supplied late and was not required to answer the questions. There were 6 questions on participants’ characteristics, including sex, county of residence, time spent in the region, projected county for professional settlement, number of children, and sources of EH information. The question of age was not asked, as the participants in this year’s class were all aged between 24 and 26 years. The participants’ data were collected and selected to be comparable with a barometric survey evaluating EH perceptions, knowledge, and behaviors in the regional population [23]. In addition, there were 2 EH knowledge questions to identify which of the suggested organizations is responsible for EH at the regional level and one Boolean question to indicate if they are familiar with the following mapping tools available to gather EH information on French territories: Rezone, Geodes, Sirsé, Atlasanté, Recosanté, ATMO-France, and RNSA [24-30]. These questions were asked only in the pretest questionnaire.

There were 20 questions related to EH to rate the participants’ knowledge for each of the 19 following EH risks from 1 (poor) to 5 (excellent): outdoor air quality, indoor air quality in buildings, noise, soil quality, radon, carbon monoxide, bathing water quality, tap water quality, Legionnaire disease, endocrine disruptors, lead, other heavy metals (cadmium, aluminum), electromagnetic waves, pesticides, nanomaterials, allergenic plant pollens, vector-borne diseases (eg, Chikungunya, Zika, yellow fever, malaria), substandard housing, and the first 1000 days of life concept. Finally, there was 1 question on the web-based reporting of EH risks to the health authorities.

There were 2 questions on behavior, as defined by the more recent version of the Kirkpatrick model [20]. Participants were asked to select how much they consider the possible effects of the environment on their health and on their patients’ health (consumption, protection, vigilance) on a scale from 1 (not at all) to 5 (totally).

There were 2 questions about their medical thesis: one asking if they would like to work on an EH thesis and one to invite them to write down their ideas for a thesis about EH.

There was 1 question about their general satisfaction level with the e-learning (completely satisfied, somewhat satisfied, don’t know, somewhat not satisfied, not at all satisfied) and 1 question to rate their level of satisfaction for each module. These questions were only available in the posttest questionnaire. Finally, there was 1 general-comments section.

The data from each of the pre- and posttest questionnaires were collected from the university’s secure website, reported into a spreadsheet, matched for each participant, and anonymized for statistical analysis. Participants who did not complete the posttest questionnaire were excluded from the final analysis.

### Data Analysis

The data were analyzed by a statistician from the research department of the Montpellier University Hospital Center. The responses given on Likert scales were analyzed as quantitative data by calculating a before versus after difference (Δ) for each question; these Δ values are described using the mean with SD and range (minimum to maximum values). Quantitative variables are described using mean and SD. For the qualitative questions, we calculated the numbers and percentages of responses.

We used paired statistical tests to compare the responses before and after the e-learning and to compare the Δ value of one question with those of other questions; the paired Student *t* test was used for comparisons of quantitative variables and the McNemar *χ^2^* test was used for comparisons of qualitative variables. We controlled the α risk at .05 for each family of tests (knowledge, behavior) using the Hochberg method. There was no imputation of missing data. The data were analyzed using SAS Enterprise software Guide 8_2.

### Ethical Considerations

The need for informed consent from participants was deemed unnecessary according to national regulations; evaluation of changes in practices brought about by training medical or paramedical staff for research purposes is considered noninterventional in France and therefore does not require an ethical review or approval [31].

Ethics committee approval was not required for this study according to General Data Protection Regulation recommendations. All data for this study are stored on a hard disk protected by secure authentication. Participant data were anonymized. Participants were not compensated to take part in this program, which was valued as part of their general medicine curriculum.

## Results

### Participants

**Figure 1:**
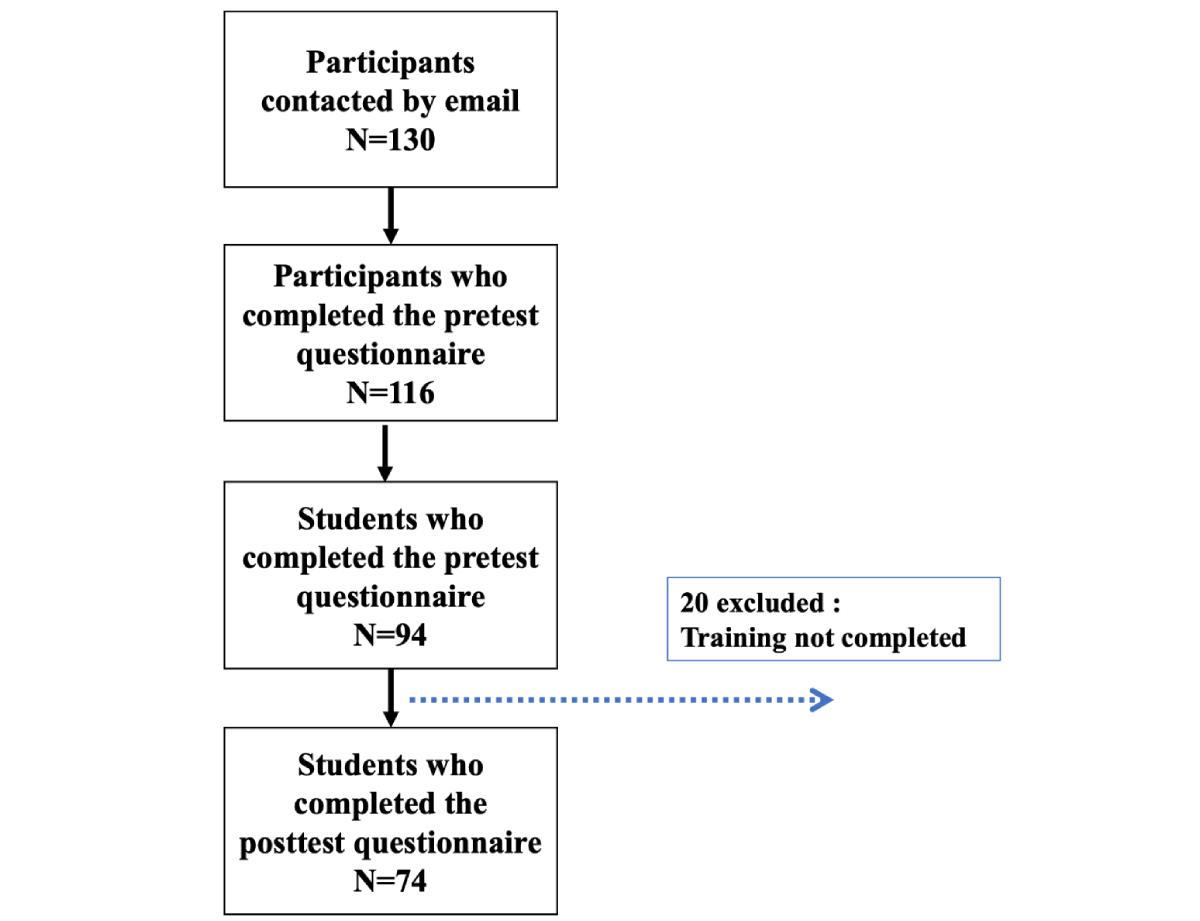
Flowchart of participation.

Of the 94 participants who completed the pretest questionnaire, 20 did not complete the posttest questionnaire; thus, 74 participants completed both the pre- and posttest questionnaires (Figure 1). The study was carried out from July 21, 2021, to September 5, 2021 (47 days) to complete the training course and answer the questionnaires. The sociodemographic characteristics of the participants are presented in Table 2. The CONSORT 2010 checklist of information to include when reporting a pilot or feasibility trial is provided in Multimedia Appendix 2.

The mean initial EH knowledge score of the participants was 2.0 (SD 0.8) out of 5. Participants initially rated their awareness of the effects of the environment on their own health at an average of 2.9 (SD 0.9) and their awareness of the effects of the environment on their patients’ health at an average of 2.6 (SD 0.9), which are both also based on a maximum total score of 5.

**Table 2:** Participants’ sociodemographic characteristics (N=74).

Sociodemographic characteristics		Participants, n (%)
Female sex		50 (67.5)
**County of residence**		
	Hérault	34 (46)
	Gard	19 (25.7)
	Pyrénées-Orientales	12 (16.2)
	Aveyron	4 (5.4)
	Aude	3 (4.0)
	Lozère	2 (2.7)
**Projected county for professional settlement**		
	I don’t know yet	34 (46)
	Hérault	17 (23)
	Gard	4 (5.4)
	Pyrénées-Orientales	3 (4)
	Another county	5 (6.8)
	Aude	5 (6.8)
	Lozère	2 (2.7)
**Time lived in Occitanie, France**		
	<1 year	25 (33.8)
	>1 year	49 (66.2)
**Number of children**		
	None	73 (98.6)
	1 or more	1 (1.4)
**EH^a^ information sources**		
	Press/radio/TV	55 (74.3)
	Official website (Ministry of Health/regional health authority)	34 (45.9)
	Relatives (friends/family)	36 (48.6)
	Health professionals	31 (41.9)
	I don’t know where to begin	18 (24.3)
	University	24 (32.4)
	Encyclopedic website	17 (23)
	Website or public forum	9 (12.2)
	Environmental protection association	7 (9.5)
	I am not interested	2 (2.7)
**Knowledge of mapping tools**		
	REZONE	4 (5.4)
	RecoSanté	6 (8.1)
	RNSA^b^	4 (5.4)
	Géodes	4.1 (3)
	ATMO^c^	6.8 (5)
	AtlaSanté	2 (2.7)
	SIRSé^d^	1 (1.4)

^a^EH: environmental health.

^b^RNSA: Réseau National de Surveillance Aérobiologique.

^c^ATMO: Fédération des Associations Agrées de Surveillance de la Qualité de l'Air.

^d^SIRSé: Système d'Information Inter-Régional en Santé.

### Satisfaction

The overall average satisfaction score was 4.0 (SD 0.9) out of 5. The average satisfaction score for each of the modules 1 to 4 was 4.0 (SD 0.9), 4.0 (SD 0.7), 4.5 (SD 0.6), and 3.5 (SD 1.3), respectively. For the qualitative assessment of posttest satisfaction, 94.6% (70/74) of participants indicated being satisfied, including 35.1% (26/74) completely satisfied and 59.5% (44/74) fairly satisfied; 5.4% (4/74) were fairly dissatisfied; and none declared being completely dissatisfied. However, 23% (17/74) of the participants declared that they had not been able to access module 4.

### Knowledge

**Figure 2:**
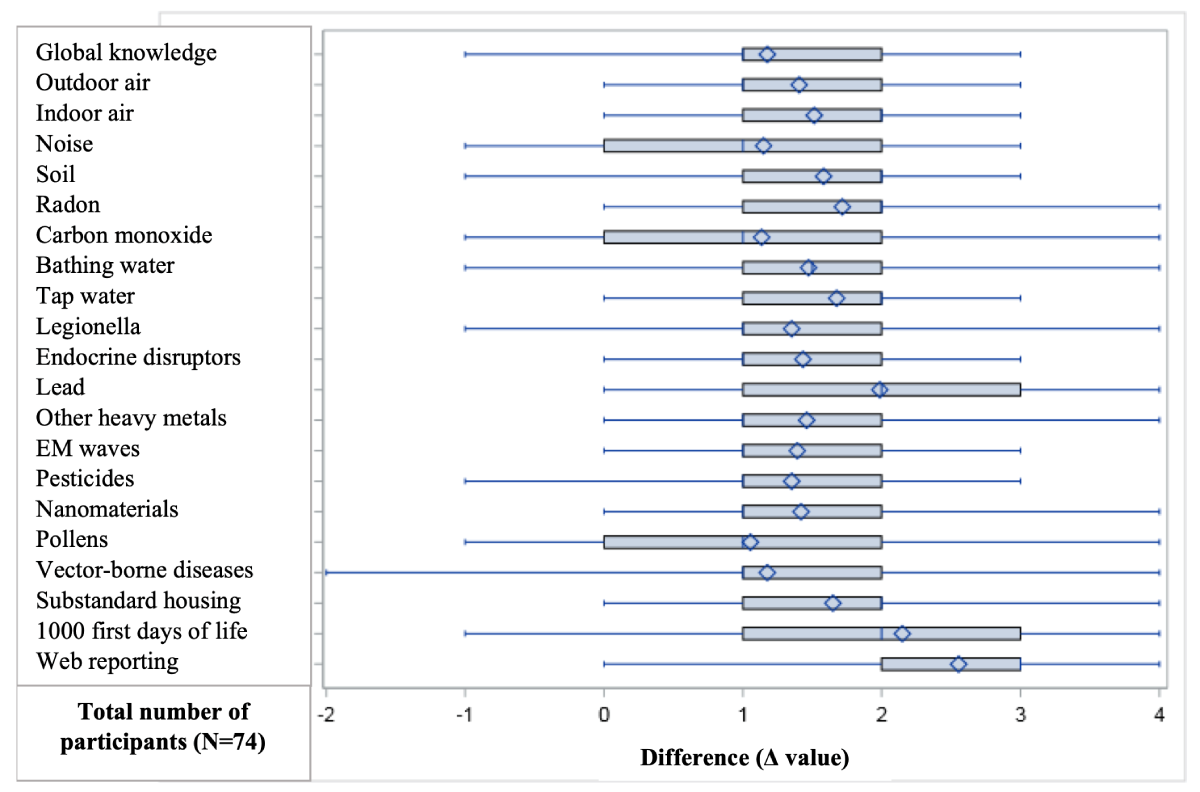
Differences in self-assessed knowledge scores before and after the course. The diamonds correspond to means, the boxes indicate the IQR, and the vertical bar indicates the median. The thin lines correspond to the extent of the difference. A paired Student *t* test was performed for each of the questions; the results were only considered statistically significant if they met the Hochberg procedure for α risk control. The results were all significant at *P*<.001. EM: electromagnetic.

There was a statistically significant increase in the knowledge of participants for all the environmental items studied (Figure 2), with an average increase of 30% (+1.5/5, SD 0.9) points for all questions (raw *P*<.001; all questions were considered to show a statistically significant difference at α=.05 after applying the Hochberg method).

Knowledge of 3 environmental items (lead, the first 1000 days of life, and web reporting) improved significantly more than the others (*P*=.005). The question on web reporting showed the greatest improvement, with an average improvement of 2.7/5 (SD 1.1) points (*P*<.001), representing an increase of 1.1/5 (SD 0.2) points on average compared to the increase in knowledge for the other questions.

The items for which the increase was less important were carbon monoxide, pollen from allergenic plants, and vector-borne diseases (Δ value of 1.1 or 1.2). The initial level of knowledge was higher by 0.7/5 (SD 0.3) points for these items.

No difference in the improvement of knowledge scores was found among participants according to their sociodemographic characteristics such as sex, children, and place of working or living.

The detailed pre- and posttest responses to Likert-scale questions on knowledge are presented in Multimedia Appendix 3.

### Behavior

**Figure 3:**
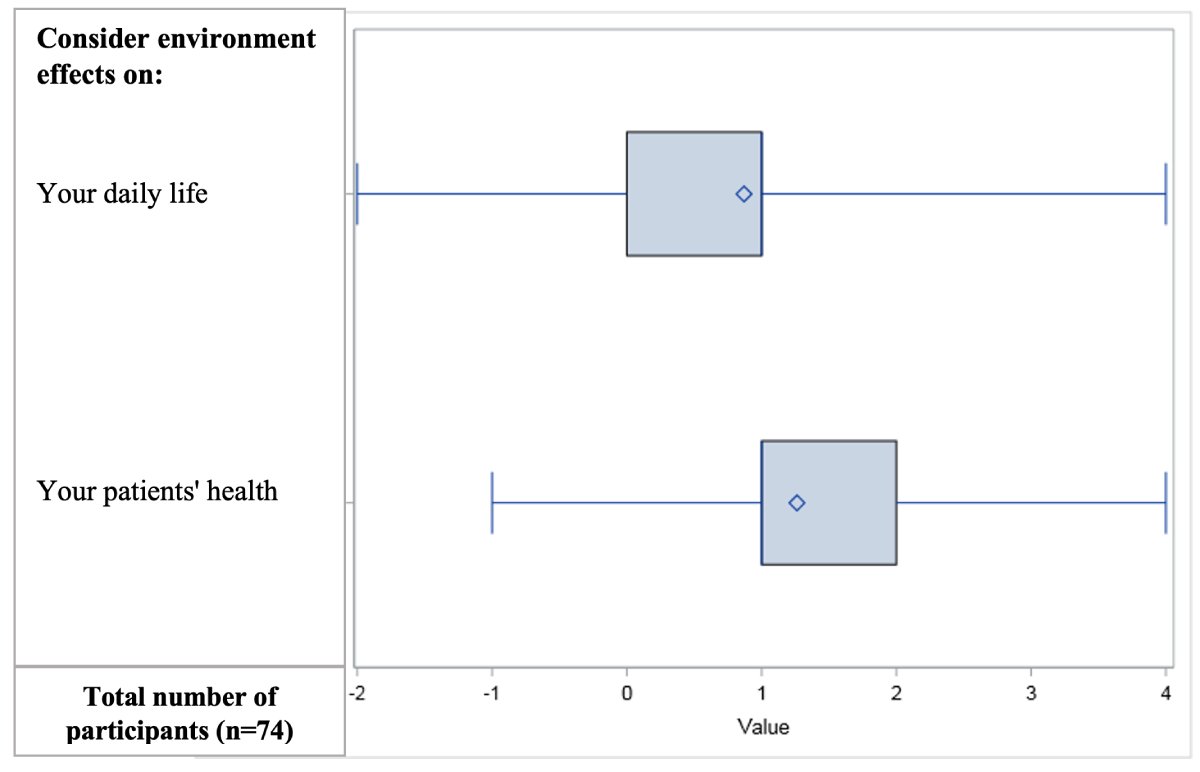
Differences in self-assessed behavior scores before and after the course. The diamonds correspond to the means, the boxes indicate the IQR, and the vertical bar indicates the median. The thin lines correspond to the extent of the difference (Δ values). A paired Student *t* test was performed for each of the questions; the results were only considered statistically significant if they met the Hochberg procedure for α risk control. The results were all significant (family *P*<.05).

There was a statistically significant increase in EH behaviors with an increase of 18% (+0.9/5 points, SD 0.4; *P*<.001) for participants considering EH-related factors in their own health and of 26% (+1.3/5 points, SD 0.5; *P*<.001) for considering EH with respect to their patients’ health (Figure 3). There was no significant increase in the number of residents declaring that they would write a thesis on EH (*P*=.07). After the training, 68.9% (n=51) of the 74 participants stated that they did not know if they wished to focus their thesis on EH, 8.1% (n=6) had answered “yes,” and 22.9% (n=17) answered “no.” Among those who answered “no” or “I don’t know,” 29.4% (20/68) described ideas for a thesis related to EH but did not know how to start. No differences in improvement were found according to participants’ sociodemographic characteristics

Detailed pre- and posttest responses to the Likert-scale questions on behavior are presented in Multimedia Appendix 3.

## Discussion

### Main Findings

The participants’ knowledge improved significantly overall and for each of the 21 environmental items studied. These items were chosen because they are easily identifiable in primary care and have a negative impact on population health [32]. The level of EH knowledge of practicing GPs is also known to be low due to their lack of training and information on the topic [33,34]. In our study, 74.3% of participants obtained information on this subject via the mainstream media, which is similar to the rate of 83.1% of citizens in Occitanie [35]. However, 45.9% of the participants of this study also used official websites to obtain this information, compared with only 18% of citizens. Only 32.4% of the participants indicated that their knowledge on this topic came from the university and 24.3% stated that they “didn’t know where to start” obtaining such knowledge. Acting on these proportions would require EH to be taught as part of the initial training. A course designed for students in their second or third year of medicine by the French Conference of Deans was deployed in medical faculties starting in 2023.

The average progress made by our participants was greater for items where their initial level of knowledge was low compared to that of topics for which the initial knowledge level was high. This expected result suggests that an initial assessment of the participants’ knowledge could facilitate providing a personalized course, prioritizing the areas of knowledge that are the least mastered, such as lead poisoning, the concept of the first 1000 days, and the web reporting of environmental pathologies to health authorities [6,14].

Self-reported behaviors showed significant improvement after the course in terms of considering the impact of the environment on their own health and the health of their patients. This suggests that the PCEH course has the potential to engage GP residents in actions on the environment, which is a promising finding given that EH will be a compulsory part of the additional year of the French GP internship [36]. Writing a medical thesis in EH would be further proof of the residents’ interest in this area. However, 68.9% of the residents included in this study stated that they did not know whether they wanted to do a thesis in EH and there was no significant increase in this proportion after the course. This question may have been asked at too early a stage in their studies. The participants were only in their first year of medical school and still had another 5 years until they need to defend their medical thesis. This will change with the introduction of junior doctors in general practice, which will reduce this period to 3 years instead of 6 years.

Further studies will be also needed to assess whether the change in behavior reported by the participants is taking place in their personal and professional lives (stage 4–*results* of the Kirkpatrick model). Achieving this level remains a challenge for health educators [37]. Indeed, this was the main motivation for designing this pilot study (ie, to prepare for the implementation of the junior doctoral year integrating EH). These findings will therefore help us to develop a higher-quality study to evaluate the impact of the PCEH course.

### Limitations

The satisfaction rate of the e-learning among participants was 94.6%. Satisfaction rates of training courses delivered in the same format often exceed 70% [16]. Overall satisfaction with the course (e-learning and face-to-face training) was not assessed. Our priority in this study was the e-learning, as it is intended to be duplicated every year. Participants were less satisfied with module 4, Communication. This may be linked to the fact that 23% of the participants declared that they had not been able to access this module, as it took longer to produce and was published after the other modules.

The consistent improvement for all environmental knowledge items and related behaviors validates the two corresponding levels of the Kirkpatrick model.

The main limitation of this study concerned the self-scaling nature of the knowledge and behaviors reported by the participants. Selection and social desirability biases may have influenced the responses. As stated in the Study Design subsection of the Methods, a recent version of the Kirkpatrick model has added that the behavior level refers to the “processes and systems that reinforce, encourage, and reward performance of critical behaviors on the job” [20]. We assumed that taking greater account of EH for their own health and for the health of their patients could be seen as a catalyst for applying what had been learned in the PCEH course. This was also why we asked participants about their desire to write their medical thesis on EH.

A hetero-assessment would not have made it possible to simply assess all of the knowledge areas of this detailed training course. This was also a pilot study designed to test the training and guide future updates to improve e-learning.

A control group could have increased the internal validity of the results and limited possible bias. This could have been achieved by comparing two classes from different universities. This could be considered in the future with a view to the junior doctor year, as many faculties have not yet designed an EH course for GPs.

A more balanced and larger sample size would have been required if we wanted to show any influence of certain characteristics of our participants on their commitment to EH. The barometer carried out on the regional population identified the number of children as a factor favoring their commitment to EH [23]. Our sample was not superimposable with this population because 81% of our participants had no children, which is consistent for first-year residents, who are usually aged between 24 and 26 years and the average age at which French female doctors have their first child is 30 years [38].

The number of participants lost to follow-up was high (32/94, 28%) and may have induced a motivation bias. This can be explained by the short duration of the entire training course (47 days) over a summer holiday period. We tried to limit this by using an asynchronous and anonymous self-assessment questionnaire.

Finally, we noted a regression in scores after training for a minority of participants, which was likely due to a Dunning-Kruger effect [39]. For the training, some participants recognized their ignorance, causing their score to drop in the posttest as a loss-of-confidence effect.

### Conclusions and Perspectives

The results from this pilot study will be used to improve the existing e-learning itself as well as the study design of the planned EH modules to be created. In 2026, the French general medicine curriculum will include an additional year of junior doctor status. EH will be one of the new themes taught during this year and we plan to disseminate this training across other medical universities of France. This study was an opportunity to test this training with a view to more advanced studies (clinical impact) in pedagogy.

Developing a training program in EH is in line with the new training curriculum for GPs, but also with the evolution of primary care in France that is largely reorganizing itself toward coordinated practice. By 2023, such coordinated practices represented 2251 multiprofessional health houses, 455 multiprofessional health centers, and 389 territorial professional health communities, although they did not exist before 2007 [40,41]. These types of coordinated practices must be structured around a health project. Incorporating an EH diagnosis could enable coherent action to be taken on the health determinants of the populations in the areas in which they operate as part of a population-based approach [4,42]. This is in line with the “scale up actions on environment, climate change and health three-step framework”: data-based situation assessment, definition of targets and selection of actions, and implementation monitoring [43]. The PCEH course could then be assessed at level 4 of the Kirkpatrick model based on concrete actions implemented in the field for the prevention and care of patients.

## Appendix

Multimedia Appendix 1 Study questionnaire.

Multimedia Appendix 2 CONSORT (Consolidated Standards of Reporting Trials) 2010 checklist of information to include when reporting a pilot or feasibility trial.

Multimedia Appendix 3 Pre- and posttest responses to Likert-scale questions on knowledge and behavior.

## Abbreviations

CONSORT: Consolidated Standards of Reporting Trials
EH: environmental health
GP: general practitioner
PCEH: Primary Care Environment and Health
SPES: Soins Primaires Environnement et Santé
WHO: World Health Organization

## Data Availability

The data sets generated during and/or analyzed during this study are available from the corresponding author on reasonable request.
