# Supplementary material for: The Primary Care and Environmental Health e-Learning Course to Integrate Environmental Health in General Practice: Before-and-After Feasibility Study
Source: JMIR Form Res. 2024 May 9;8:e56130. doi: 10.2196/56130 (PMC11117128; doi:10.2196/56130)
Supplement: Multimedia Appendix 1 [file formative_v8i1e56130_app1.docx]

**Appendix 1**

**Pre-test questionnaire**

1. Scale your general knowledge level about environmental health (from 1 Poor to 5 Excellent).

Scale your knowledge level for each of the following environmental health parameters (from 1 Poor to 5 Excellent):

1. Outdoor air quality.
2. Indoor air quality in buildings (homes, schools, offices)
3. Noise.
4. Soil quality.
5. Radon
6. Carbon Monoxide.
7. Bathing water quality.
8. Tap water quality.
9. Legionnaire's disease
10. Endocrine disruptors.
11. Lead.
12. Other heavy metals (cadmium, aluminium).
13. Electromagnetic waves.
14. Pesticides.
15. Nanomaterials.
16. Allergenic plant pollens.
17. Vector-borne diseases (Chikungunya, Zika, yellow fever, malaria, etc).
18. Substandard housing.
19. The first 1000 days of life concept.
20. Web reporting of an environmental health risk.
21. How do you keep yourself informed about environment risks? (Several answers accepted)

- In the press, radio, TV (including on their websites).

- On websites or public forums.

- On encyclopaedic sites such as Wikipedia.

- On official websites such as the Ministry of Health or the ARS.
- With your relatives (family, friends).

- With health professionals (doctors, etc.).

- With patient, consumers, and environmental associations.

- At university, in courses (e.g., medical school).

- I don't keep myself informed (I don't know where to start).

- I don't keep myself informed (I'm not interested).

1. Which of the following organisations is responsible for environmental health at regional level? (Only one answer accepted)

- The Regional Health Agency (ARS)
- The Regional Directorate for the Environment, Planning and Housing (DREAL).

- The Regional Medical Council.

- The Regional Unions of Health Professionals.
- The Federation of Territorial Hospital Groups.

- The local university of medicine.

1. Are you familiar with the following mapping tools (Yes/No)

- REZONE

- GEODES

- SIRSE

- ATLASANTE

- RECOSANTE

- ATMO website

- RNSA website

1. In your daily life, scale how much you consider the possible effects of the environment on your health (consumption, protection, vigilance)? (From 1 Poor to 5 Excellent).
2. In your professional life, scale how much you consider the possible effects of the environment on your patients’ health (consumption, protection, vigilance) (from 1 Poor to 5 Excellent)
3. The website signalement.social-sante.gouv.fr/ allows to report events at risk of environmental health, such as drugs sides effects reporting website (pharmacovigilance)

-Yes

-No

-I don't know

1. I am:

-A woman

-A man

1. In which French county do you currently live?

-Ariège (09)

-Aude (11)

-Aveyron (12)

-Gard (30)

-Haute-Garonne (31)

-Gers (32)

-Hérault (34)

-Lot (46)

-Lozère (48)

-Hautes-Pyrénées (65)

-Pyrénées-Orientales (66)

-Tarn (81)

-Tarn-et-Garonne

-Another county

1. In which department do you plan to settle as a general practitioner?

-Ariège (09)

-Aude (11)

-Aveyron (12)

-Gard (30)

-Haute-Garonne (31)

-Gers (32)

-Hérault (34)

-Lot (46)

-Lozère (48)

-Hautes-Pyrénées (65)

-Pyrénées-Orientales (66)

-Tarn (81)

-Tarn-et-Garonne

-Another county

-I don’t know

1. Among the people living in your household, how many children are under 16 years old?
2. How long have you lived in Occitanie (Region of France)?
3. Would you like to work on an environmental health thesis?

-Yes

-No

-I don’t know.

1. Please write down here if you have ideas for a thesis topic about environmental health (free text)

**Post-test questionnaire**

1. Scale your general knowledge level about environmental health (from 1 Poor to 5 Excellent).

Scale your knowledge level for each of the following environmental health parameters (from 1 Poor to 5 Excellent):

1. Outdoor air quality.
2. Indoor air quality in buildings (homes, schools, offices)
3. Noise.
4. Soil quality.
5. Radon
6. Carbon Monoxide.
7. Bathing water quality.
8. Tap water quality.
9. Legionnaire's disease
10. Endocrine disruptors.
11. Lead.
12. Other heavy metals (cadmium, aluminium).
13. Electromagnetic waves.
14. Pesticides.
15. Nanomaterials.
16. Allergenic plant pollens.
17. Vector-borne diseases (Chikungunya, Zika, yellow fever, malaria, etc).
18. Substandard housing.
19. The first 1000 days of life concept.
20. Web reporting of an environmental health risk.
21. The website signalement.social-sante.gouv.fr/ allows to report events at risk of environmental health, such as drugs sides effects reporting website (pharmacovigilance).

- Yes

- No

- I don’t know

1. Would you like to work on an environmental health thesis?

- Yes

- No

- I don’t know

1. Please write down here if you have ideas for a thesis topic in environmental health (free text).
2. After following the PCEH training, scale how much you consider the possible effects of the environment on your health (consumption, protection, vigilance)? (From 1 Not at all to 5 Totally)
3. After following the PCEH course, scale how much you consider the possible effects of the environment on your patients' health (consumption, protection, vigilance) (from 1 not at all to 5 totally).
4. Concerning the E-learning of the PCEH course, please rate your satisfaction level with the e-learning (only one answer possible):

-Completely satisfied

-Somewhat satisfied

-Don't know.

-Somewhat not satisfied

-Completely not satisfied

1. General comments on E-learning (free text)
2. Any comments on modules I, II, III, IV?

Regarding the different modules of the PCEH course, please rate your level of satisfaction (from 1 poor to 5 Excellent) :

1. Module I Introduction
2. Module II Population approach
3. Module III Clinical cases
4. Module IV Communication.
5. Do you have any general comments/suggestions for the PCEH course (free text)
