# Supplementary figures and images for: The Primary Care and Environmental Health e-Learning Course to Integrate Environmental Health in General Practice: Before-and-After Feasibility Study
Source: JMIR Form Res. 2024 May 9;8:e56130. doi: 10.2196/56130 (PMC11117128; doi:10.2196/56130)

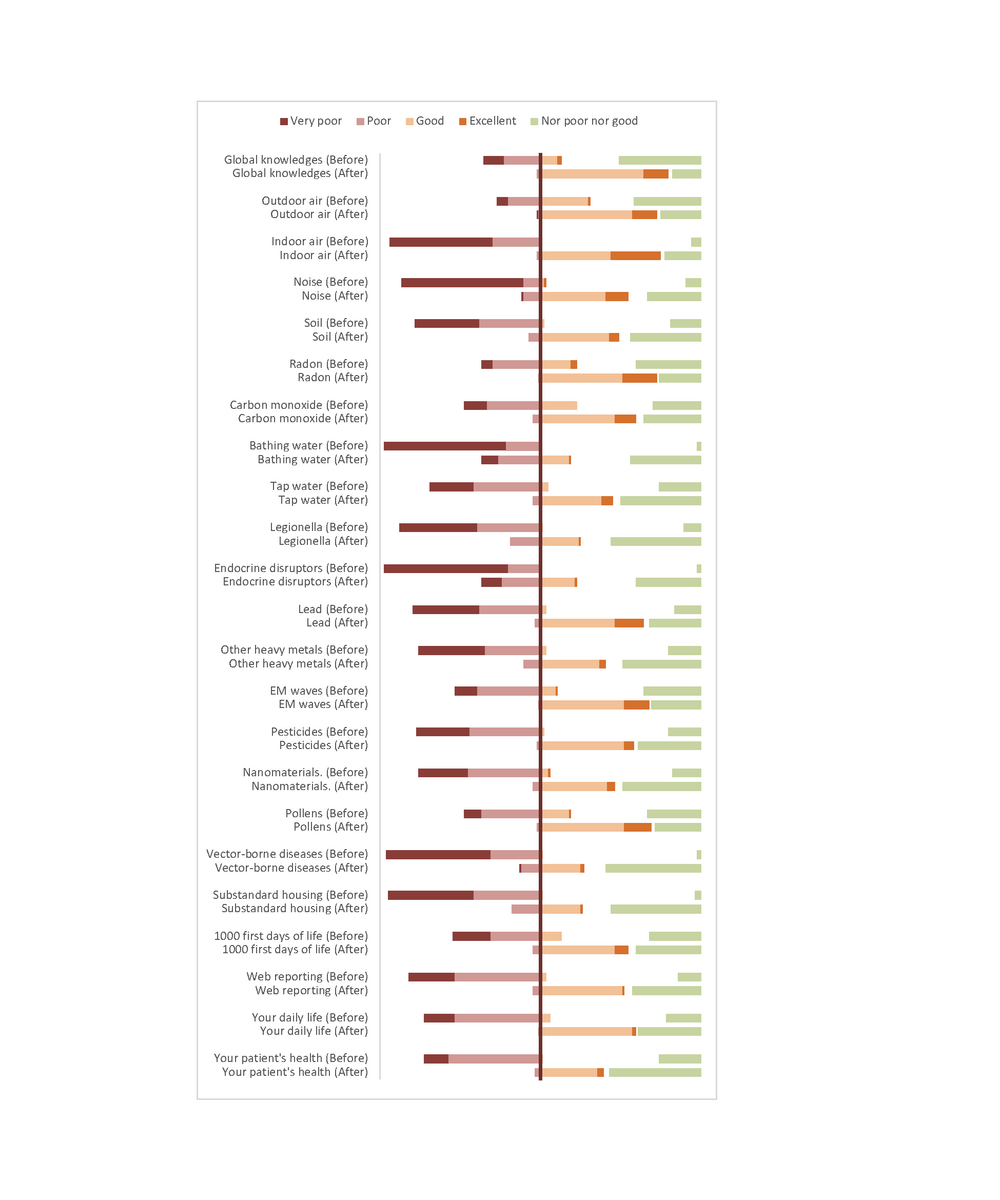

Supplement: Multimedia Appendix 3 [file formative_v8i1e56130_app3.png]
